# Cooperation of Oligodeoxynucleotides and Synthetic Molecules as Enhanced Immune Modulators

**DOI:** 10.3389/fnut.2019.00140

**Published:** 2019-08-27

**Authors:** Shireen Nigar, Takeshi Shimosato

**Affiliations:** ^1^Department of Nutrition and Food Technology, Jashore University of Science and Technology, Jashore, Bangladesh; ^2^Department of Biomolecular Innovation, Institute for Biomedical Sciences, Shinshu University, Nagano, Japan

**Keywords:** ligands, molecule, ODN, synergy, TLR

## Abstract

Unmethylated cytosine–guanine dinucleotide (CpG) motifs are potent stimulators of the host immune response. Cellular recognition of CpG motifs occurs via Toll-like receptor 9 (TLR9), which normally activates immune responses to pathogen-associated molecular patterns (PAMPs) indicative of infection. Oligodeoxynucleotides (ODNs) containing unmethylated CpGs mimic the immunostimulatory activity of viral/microbial DNA. Synthetic ODNs harboring CpG motifs resembling those identified in viral/microbial DNA trigger an identical response, such that these immunomodulatory ODNs have therapeutic potential. CpG DNA has been investigated as an agent for the management of malignancy, asthma, allergy, and contagious diseases, and as an adjuvant in immunotherapy. In this review, we discuss the potential synergy between synthetic ODNs and other synthetic molecules and their immunomodulatory effects. We also summarize the different synthetic molecules that function as immune modulators and outline the phenomenon of TLR-mediated immune responses. We previously reported a novel synthetic ODN that acts synergistically with other synthetic molecules (including CpG ODNs, the synthetic triacylated lipopeptide Pam_3_CSK_4_, lipopolysaccharide, and zymosan) that could serve as an immune therapy. Additionally, several clinical trials have evaluated the use of CpG ODNs with other immune factors such as granulocyte-macrophage colony-stimulating factor, cytokines, and both endosomal and cell-surface TLR ligands as adjuvants for the augmentation of vaccine activity. Furthermore, we discuss the structural recognition of ODNs by TLRs and the mechanism of functional modulation of TLRs in the context of the potential application of ODNs as wide-spectrum therapeutic agents.

## Introduction

The immune system provides biosecurity against aggression by pathogens. The human body maintains impediments to prohibit entrance by microbes. Innate and adaptive components are part of the immune system. Innate immune responses provide a prompt response by the body. Innate immune reactions are mediated by Toll-like receptors (TLRs), a representative, evolutionarily conserved group of proteins that are able to induce innate immune reactions, particularly against bacterial infections, through the recognition of pathogen-associated molecular patterns (PAMPs) (1, 2), to recognize and eradicate invading organisms (3). The germline-encoded pattern recognition receptors (PRRs) recognize conserved molecular patterns that activate the innate immune system. This response is coordinated by specialized cells such as basophils, dendritic cells (DCs), eosinophils, macrophages, monocytes, natural killer (NK) cells, neutrophils, and NKT cells (4). PRRs bind to molecular identification molecules, fundamental structural molecules, or historical segments that are preserved among microbes. This provides the innate immune system with the ability to rapidly counter a comprehensive scope of contagious agents that contact the dermis and mucous membranes.

TLRs are an important family of PRRs that are activated by PAMPs expressed by bacteria, viruses, fungi, and protozoa (5, 6). PAMPs are the key molecules that mediate microbial destruction by processes such as mobilization of phagocytes to infected tissues and microbial killing. TLR family members are classified into two types: cell membrane receptors and endosome-associated receptors. Here, lipoprotein is derived from gram-positive bacteria and induces signaling pathways through TLR2 and other receptors (7, 8). Lipopolysaccharide is a cell wall component of gram-negative bacteria that induces signaling through TLR4 (9). Flagellin (a TLR5 agonist) is derived from the cell wall of *Saccharomyces cerevisiae*. Zymosan, an extract from *S. cerevisiae*, is recognized by another heterodimer, TLR2/6. These TLR agonists act as potent immune stimulators that activate adaptive immune responses as well as recognize microbial components such as lipids, lipoproteins, and flagella. Different types of TLR have different recognition sites. TLR3 recognizes double-stranded RNA, and TLR7/8 recognizes single-stranded RNA. On the other hand, the recognition site of TLR9 is single-stranded synthetic oligodeoxynucleotides (ODNs) or viral/microbial DNA. TLR9 also recognizes unmethylated cytosine-guanine dinucleotide (CpG) motifs (10–13) expressed in the intracellular vesicles of prokaryotic cells (e.g., endoplasmic reticulum, endosomes, and lysosomes).

TLRs are part of a universal group of molecules that consists of extracellular leucine-rich repeats and a cytoplasmic Toll interleukin-1 receptor domain (14). TLRs recognize PAMPs and have specific immune functions. TLR activation initiates and maintains innate and adaptive immune pathways in association with memory function (15). Adapters induce signaling cascades that terminate in the stimulation of nuclear factor kappa B, mitogen-activated protein kinase, and interferon (IFN) regulatory factors 1, 3, 5, and 7 (16). Collectively, these transcription factors induce a variety of cytokines, and chemokines, some of which regulate cellular proliferation, growth, and maintenance. Thus, TLRs are an important group of receptors through which the innate immune system recognizes invasive microorganisms. Here, we discuss the synergistic activities of TLR1/2, TLR4, TLR2/6 with synthetic ODNs in the immune system. Furthermore, we discuss the immune synergistic phenomenon of TLRs in the context of the potential application of ODNs as therapeutic agents.

## Oligodeoxynucleotides as Immune Modulators

### CpG ODNs

ODNs containing CpG motifs trigger host defense mechanisms that involve both innate and adaptive immune responses. TLR9 recognizes viral/microbial DNA containing CpG motifs, triggering alterations in the cellular redox balance and the induction of cell signaling pathways, including mitogen-activated protein kinase and nuclear factor kappa B (17). Bacterial DNA serves as a PAMP, which is recognized by the vertebrate immune system for coordination of immune responses correlated with immunity (18). CpGs are extremely prevalent in prokaryotic DNA, but rare in eukaryotic DNA (19, 20). Overall, CpG hexamer motifs contain one or more CpG-deoxynucleotides, and the number, position, spacing, and surrounding bases of these motifs mediate their immunostimulatory activities (21–23). These CpG-hexamer motifs also have species-specific activity, which is determined by their structural features and length (18, 24, 25). Another research group explored whether bacterial DNA combines with other bacterial products to trigger the secretion of interleukin (IL)-6, IL-12, IFN-γ, and IgM (26). CpG motifs of synthetic ODNs stimulate B cells (18, 27), NK cells (28, 29), and specialized antigen-presenting cells (APCs) to multiply and/or secrete a variety of cytokines, chemokines, and immunoglobulins (30–32). CpG class A (CpG-A) is particularly effective at stimulating NK cells and inducing production of IFN-α by plasmacytoid dendritic cells (pDCs), whereas CpG class B (CpG-B) is an especially potent B-cell activator. CpG-driven immune activation aggravates inflammatory tissue deterioration, stimulates the advancement of autoimmune disorders, and enhances lethal shock (33–37).

CpG DNA has been sought after as a therapeutic agent and adjuvant immunotherapy for the treatment of cancer, asthma, allergies, and infectious diseases, but this agent frequently involves phosphorothioate or chemical modification. Such modification may lead to imperfections owing to integral toxicity combined with an ephemeral anticoagulant effect, induction of the complement cascade, or inhibition of a primary fibroblast growth component attached to surface receptors due to non-specific protein binding (38). Therefore, CpG DNA can activate Th1 cytokine production, which stimulates a cytotoxic T-cell response with increasing Ig production; this has been observed in the therapy of a wide spectrum of infections, including viral infections and inflammatory disorders (21, 39–41).

Several classes of CpG ODNs exist and are based on structural attributes and immunomodulatory actions. Examples include CpG-A (also known as type D) (42), which induces secretion of IFN-α and stimulates pDC maturation, and monomeric CpG-B (or type K) (42), which induces production of tumor necrosis factor (TNF)- α, promotes pDC maturation, and fully stimulates B cells. Lastly, the dimeric CpG class C (CpG-C) incorporates elements of both CpG-A and CpG-B, albeit with intermediate strength. Different CpG ODNs are assembled CpG-A or CpG-B/C with a phosphorothioate backbone to avoid degradation by serum nucleases and to augment *in vitro* and *in vivo* activity. Apart from CpG-A, CpG-B, and CpG-C, some researchers have suggested another unique class, P-class CpG ODN (CpG-P) (41), which can induce IFN-α production more than class C ODNs due to inclusion of two palindromic sequences. Therefore, synthetic CpG ODNs are considered to be promising immunomodulators (40).

### Novel Synergistic ODNs

The immunosynergistic effects of ODNs have been established in ODN research. Initially, research was conducted on the immunomodulatory (43), immunosuppressive (44, 45), and immunostimulatory (46) effects of ODNs. In 2017, Nigar et al. explored a novel ODN (named “iSN34”) incorporated into *Lactobacillus rhamnosus*, GGATCC53108, that has synergistic effects on the immune response in CpG-induced immune activation (47). The iSN34 sequence is TTCCTAAGCTTGAGGCCT (48). Originally, we evaluated inhibitory or suppressive ODN (iODNs) with a TTAGGG motif. In 2013, Ito et al. successfully designed an effective iODN (designated “iSG3”) and revealed that iSG3 has strong immunosuppressive activity (49). As a synergistic ODN, iSN34 could be incorporated with CpG to trigger the production of IL-6 and IL-6-secreting CD19^+^ B cells. Thus, iSN34 may be an extraordinarily impressive adjuvant-mediated vaccine via Th1 that defends against intracellular pathogens, or iSN34 may provide antibody-mediated immunity compared to extracellular pathogens (47). We also conducted further investigations into the synergistic induction of IL-6 by the consolidation of iSN34 with the cell wall components of bacteria (TLR1/TLR2, TLR4) and fungi (TLR2/TLR6) (48). This review focuses on the synergistic immune effects of combining CpG ODNs with synthetic molecules associated with TLR ligands targeting inflammation and autoimmune diseases.

## Immunotherapeutic Application of ODNs and Other Synthetic Molecules

### Prevention and Treatment of Innate and Adaptive Immunity Dysfunction

Innate and adaptive immune responses are two key components of the immune system. Innate immune feedback is recognized as the body's primary defense by which non-specific cells engulf foreign organisms and molecules. Many cells of the immune system (monocytes, macrophages, DCs, neutrophils, eosinophils, basophils) along with NK cells and NKT cells (50) harmonize this response. These specialized cells bind to molecular signatures and are triggered by germline-encoded PRRs, which are fundamental receptors that recognize molecules that are conserved among microbes. This allows the innate immune system to react expeditiously to an extensive spectrum of organisms and protects the dermis and mucous membranes. In the innate immune system, TLRs are recognized as pattern recognition molecules. Alternatively, in the adaptive immune system, APCs are frequently triggered by activation of TLRs, are limited in mobilization, but maintain a remarkably antigen-specific response. This is attained through T-cell and B-cell receptors following somatic recombination. The innate immune system effectively defends humans from an immense spectrum of contaminants by relying on antecedent molecules. Moreover, the innate immune system eliminates the infectious microorganisms that trigger PRRs. Therefore, an equity between stimulation and inhibition must be maintained to provide a secure and potent immune response. Further investigation of the activity and response of the innate immune system will support the design of reliable and efficient therapeutic interventions.

In the vertebrate immune system, viral/microbial DNA that acts as PAMPs coordinates immune responses involving both innate and adaptive immunity. This stimulates the proliferation of B cells and the production of cytokines such as IL-12, IFN-γ, IL-6, and TNF-α, and of co-stimulatory molecules by monocytes/macrophages, B cells, and DCs (51). In this context, the ability of bacterial DNA to induce IL-6 is of particular concern. Immunomodulatory CpG ODNs are currently being assessed to establish the optimum induction of specific cytokine profiles and the activation of individual and mouse immune cells. In 2017, Nigar et al. demonstrated the immune synergistic effects of a combination of a non-CpG ODN (iSN34) and a CpG ODN (CpG-B). The activity of iSN34 was combined with different types of CpG-ODNs. The synergistic effects of iSN34 and CpG-B to stimulate the production of IL-6 would be an immensely effective adjuvant for Th1-mediated vaccines. The combined activity may be advantageous for the avoidance or treatment of inflammatory disorders such as rheumatoid arthritis, inflammatory bowel disease, multiple sclerosis, systemic-onset juvenile chronic arthritis, osteoporosis, and psoriasis (52–54). The synergistic effects of iSN34 and CpG ODN may have the unique ability to target the treatment of systemic inflammatory diseases.

### Use of ODNs as Immunotherapeutics Against Inflammatory Diseases

Inflammation is a vital process in response to injury or infection that occurs through a sequence of events to produce wound healing and maintenance of normal tissue homeostasis. It is a complicated process involving molecular mechanisms that recognize specific molecular patterns associated with infection or tissue injury. The inflammatory response is mediated by key regulators that are proinflammatory molecules. Inflammation involves the contribution of many enzymes and the infiltration of heterogeneous cells that modify chemokine gradients as well as the inflammatory response (55, 56).

**Figure 1:**
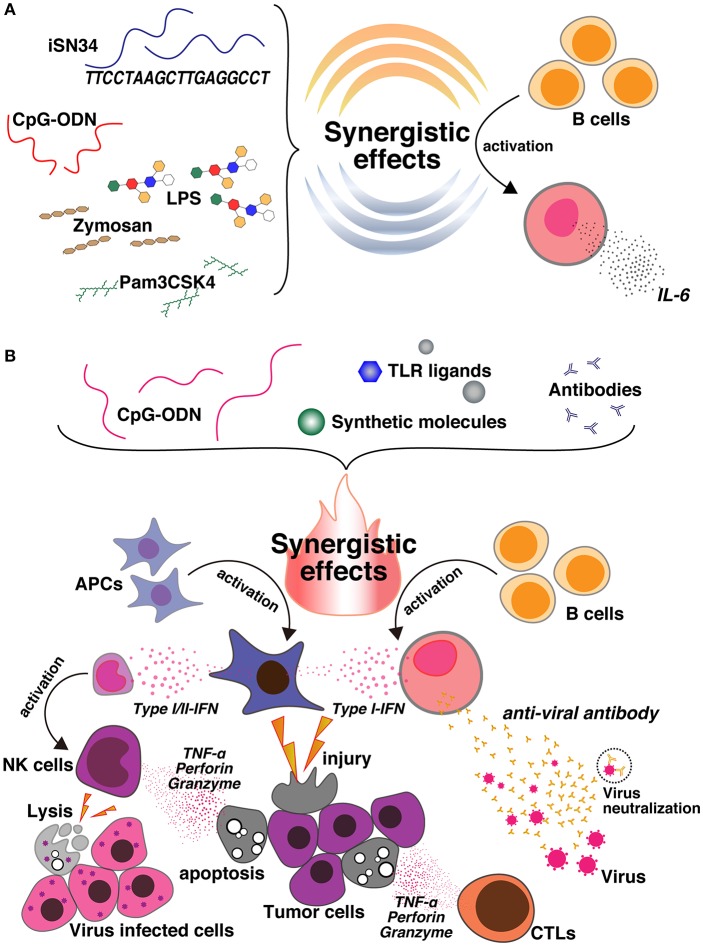
**(A)** CpG ODNs are synergistically activated with a novel ODN, iSN34, and other TLR ligands, such as Pam_3_CSK_4_ (TLR1/2), LPS (TLR4), and Zymosan (TLR2/6). This synergy enhances IL-6 induction and activates B cells. **(B)** Co-delivery of CpG ODNs and different TLR ligands, synthetic molecules, and antibodies produces an immunosynergistic response, which promotes the secretion of Type I/II-IFN cytokines and also the production of B cells. This leads to the generation of tumor-specific antibodies, which may be useful for enhancing antitumor agents, cancer vaccines, and the immunoregulatory effects against inflammatory disorders, as well as enhancing antiviral action and facilitating apoptosis. In contrast, the synergy of CpG and the synthetic molecule also activates NK cells, leads to cell lysis, and is useful for preparing vaccines against virally infected cells.

Unwanted inflammation occurs regardless of whether consolidation of TLR3/TLR9 agonists leads to IFN-α or B-cell activation or the potentially immunoregulatory function of IL-10 (57). In a model of measles virus pathogenesis, B-cell proliferation and antibody secretion are suppressed not only by maternal antibodies (58, 59), but also by T-cell responses (60–64). The main mechanism of B-cell responses includes the neutralization of live-attenuated vaccines, which reduces epitope masking and interferes with the recognition of antigens by B cells. Then, neutralizing antibodies can only produce a B-cell response when the antibody can attach to the Fcγ IIB receptor (CD32) on the B-cell surface (65). In 2018, Nigar et al. reported that association of iSN34 with TLR1/2, TLR4, and TLR2/6 induces IL-6, leading to a potent immune response (Figure 1A). They also revealed that the effects highlight the potential of TLRs, especially CD19^+^ cells, as managers of B-cell activation, which performs a vital role in antigen-autonomous evolution and immunology-induced activation of B cells. These results suggest that inflammatory diseases can perhaps be treated by targeting upstream progression in innate immune cells.

### Vaccination

#### T-Cell Vaccination

T-cell lymphomas are infrequent, but antagonistic malignancies can occur due to resistance to chemotherapy and chemotoxicity. The combination of melanoma with recurrent spontaneous CD8^+^ T-cell responses is of clinical concern. In all enrolled patients, T-cell numbers improved following vaccination with Montanite (incomplete Freund's adjuvant, IFA) as an adjuvant treatment, and marked T-cell responses, which are associated with predominant generation of effector-memory-phenotype cells, were observed. Consequently, imiquimod induced a significant increase in the number of key-memory-phenotype cells and larger percentages of CD127^+^ (IL-7R) T cells in lymphoma, whereas imiquimod and CpG-ODN synergistically induced the growth of effector CD8^+^ T cells. Another investigation explored the T-cell induction properties associated with various adjuvants (IFA, imiquimod 5%) administered by unusual routes, such as subcutaneous, intradermal, and intranasal. That study did not report any autoimmune-related reactions after introducing immunotherapy with anti-cytotoxic T-lymphocyte-associated protein 4 antibody (ipilimumab) (66). By contrast, our data emphasize that significant proportions of central memory (CM)-phenotype cells (CD45RA^−^CCR7^+^) with enhanced expression of CD127 were induced by imiquimod. Therefore, in triggering the TLR7 agonist, imiquimod may elevate memory differentiation and potential T-cell responses (67–69). Despite the interest in memory cells, insufficient synthetic vaccines have been developed for the treatment of lymphoma. The generation of memory cells may depend on the canonical Wnt pathway with β-catenin/T-cell factor-1, the mammalian target of rapamycin, and AMP-activated protein kinase signaling pathways (67–69). Here, we indicate that the combination of imiquimod and IFA synergistically leads to the enhancement of memory-phenotype cells. This article documented that the synergistic effect of imiquimod and IFA may enhance vaccine efficacy (70), which supports the idea that innate immunity may be triggered via multiple microbe-associated molecular patterns.

#### Measles Vaccines

CpG ODNs, which are generally recognized as present in viral/microbial DNA, but are unusual in mammalian DNA, enhance the innate immune response. ODNs and TLR ligands synergistically induce an immune response by triggering different signaling pathways and may affect antigen-dependent T-cell immune memory. Viruses induce type-I IFN through TLR3, TLR7, and TLR9. A combination of TLR3 and TLR9 agonists, which enhance the induction of type I IFN, was tested and found to fully activate the B-cell response after vaccination when assigned to inhibitory MeV-specific IgG. This mechanism suggests that TLR3 and TLR9 signal via the TIR-domain-containing adapter-inducing IFN-α (TRIF)/interferon regulatory factor (IRF)-3 and MyD88/IRF-7 pathways, respectively, as well as IFN-α and IFN-β, respectively. The principal tenet is that an amalgam of these agonists would result in greater induction of type-I IFN because of the synergistic effects mediated through the IFN receptor. This review suggests that the measles vaccine may lead to an optimum antibody response, even though it is reassembled with TLR3 and TLR9 agonists, which may have enormous potential for clinical control. An essential issue that remains unresolved in vaccinology is the inhibition of vaccination against transmittable diseases in humans (71–77) and animals (78–86) by maternal antibodies. In a recent review of measles vaccines in both patients and cotton rats (61), a research group discovered a relevant model of measles virus pathogenesis and affirmed that the proliferation of B cells and secretion of antibodies are suppressed by maternal antibodies (62). In addition, T-cell responses are often noticeable (60, 63, 64, 66, 69). However, the suppressive mechanisms of B-cell responses by maternal antibodies comprise the neutralization of live attenuated vaccines. The neutralization of live attenuated vaccines is not reasonable because the immune response against protein vaccines is further inhibited by maternal antibodies, and B-cell responses can also be suppressed by non-neutralizing antibodies (69) and attach to the Fcγ IIB receptor (CD32) on the surface of B cells (87).

#### DNA Vaccine in a Mouse Model of B-Cell Lymphoma

B-cell lymphoma, also known as B-cell non-Hodgkin's lymphoma (NHL), comprises 90% of NHLs, which develop from different stages of B-cell growth and development. NHL is a diverse group of disorders that have divergent ancestral, histologic, and clinical backgrounds. Patients with NHL initially receive treatment with rituximab, a monoclonal anti-CD20 antibody, as well as cyclophosphamide, hydroxydaunorubicin, oncovin, and prednisone. Looking only at lymphoma targets, anti-CD20 contributes to B-cell deficiency through the following three essential mechanisms: (i) initiation of apoptosis, in which anti-CD20 inhibits intracellular signaling pathways; (ii) initiation of complement-dependent cytotoxicity; and (iii) approach to anti-CD20 targeted B cells (generally NK cells or macrophages), thereby stimulating antibody-dependent cytotoxicity (88).

The efficacy of cancer vaccines usually depends on the essential strength of Th1 and Th2 responses prompted by APCs and DCs, respectively. Th1-driven cytotoxic T-cell responses are effective for eradicating tumor cells. However, CpG ODNs, which are universally investigated TLR9 agonists, enhance the Th1 response and likewise result in substantial levels of the anti-inflammatory Th2-promoting cytokine IL-10, which could counteract the increasing Th1 response. Furthermore, concurrent immunotherapy with CpG ODNs and IL-10 siRNAs synergistically increases immune protection through a DNA vaccine against B-cell lymphoma in a prophylactic murine model. These outcomes suggest that PAMPs can be administered to modulate TLR ligand-mediated immune stimulation precisely in DCs through the co-delivery of cytokine-silencing siRNAs, thereby boosting antitumor immunity through an idiotype DNA vaccine in a mouse model of B-cell lymphoma.

### Cancer Immunotherapy

#### Immunotherapeutics Against Lewis Lung Carcinoma

The TLR9 agonist CpG ODN has shown promise as an effective treatment for infectious disease, allergic disease and to have encouraging antitumor activity mediated by the stimulation of antitumor immunity in numerous animal models. Increasing evidence has shown significant advances in cancer immunotherapy, and numerous strategies have been developed to deliver a tumor-specific immune response (89). Here, the synergistic effect of a TLR2-neutralizing antibody and a TLR9 agonist CpG ODN was observed to advance coherent immunotherapy against tumor metastasis. The mechanism of action for this combination regimen has been investigated (90). Metastasis treatment with CpG ODNs plus an anti-TLR2 antibody synergistically suppressed and subsequently improved infiltration of NK and cytotoxic T cells, reduced the recruitment of type-2 macrophages and regulatory T cells, and reduced the expression of immunosuppressive factors together with transforming growth factor-β1, cyclooxygenase-2, and indoleamine 2,3-dioxygenase. However, in a metastatic Lewis lung carcinoma mouse model, CpG ODNs and an anti-TLR2 antibody regimen eradicated synergistic immunosuppressive tissues from the tumor environment.

Immunosuppression by the TLR2 and TLR9 combination regimen on host immune and tumor cells for controlling metastatic behavior was associated with a nominal effect on initial subcutaneously embedded tumor growth (91–94). In 2007, Krieg demonstrated that CpG ODNs have a promising anticancer immunotherapeutic ability to trigger Th1 antibodies in the innate and adaptive immune system. On the other hand, TLR2 is an inimitable member of the TLR family because it activates an immunosuppressive response *in vivo* (95). The administration of CpG ODNs also improved the frequency of NK and cytotoxic T lymphocyte (CTL) infiltration, secretion of IFN-γ, and differentiation of M1 cells, but did not reduce the number of regulatory T cells in the spleen (89). These findings show that the synergistic effects of both CpG ODNs and the TLR2-neutralizing antibody are the result of enhanced immune cytotoxicity against tumor cells and show an anti-metastatic effect.

#### Evaluation of Tumor Immunization

In this review, we discuss the synergistic activity of CpG ODNs and stimulator of interferon gene (STING)-ligand cyclic guanosine monophosphate-adenosine monophosphate (cGAMP). The STING-cGAMP interaction and CpG ODNs terminate NK cells, lead to production of IFN-γ, have similar effects as IL-12 and type-I IFNs, and are differentially controlled by IRF3/7, STING, and MyD88. The aggregation of CpG ODNs and cGAMP is an effective type-1 adjuvant that leads to robust Th1-type and cytotoxic CD8^+^ T-cell responses. In murine tumor models, researchers administered intratumorally vaccinated CpG ODNs and cGAMP synergistically, which resulted in a significantly decreased tumor size. This treatment thus functioned as an antigen-free anticancer agent. Moreover, Th1 cells play vital roles in the generation of antitumor immunity, which resulted in suitable activation and effector functions of CTLs, including IFN-γ production (96, 97). Thus, Th1 cells are the key inducers of type-1 immunity and are preeminent in phagocytic activity (98, 99). An important feature of CpG ODNs, mainly D-type CpG ODNs, is that they strongly induce both type-I and type-II IFNs, and are also rather incapable of inducing B-cell activation (42, 46). Taken together, these findings indicate that the synergistic effects induced by K3 CpG and cGAMP may lead to potent activation of NKs and induction of IFN-γ. However, these mechanisms partially rely on IL-12 and type-I IFNs. This review also illustrates that the synergistic effects of CpG ODNs and cGAMP result in a strong antitumor agent, suggesting that synergy may be advantageous for immunotherapeutic applications (100).

#### Treatment of B-Cell Chronic Lymphocytic Leukemia

B-cell chronic lymphocytic leukemia (B-CLL) is the most prevalent adult leukemia, targeting mainly older individuals in the U.S., Europe, and Australia (101). Its clinical progression involves stroma-dependent B-CLL growth within lymphoid tissue. Mongini et al. reported that high proliferator status *in vitro* was linked to diminished patient survival with immunohistochemical evidence of apoptotic cells and IL-15-producing cells proximal to B-CLL pseudo-follicles in patients' spleens. They also suggested that ODNs and IL-15 signaling may synergistically promote *in vivo* B-CLL growth. B-CLL depends on TLR9 signals, which led some researchers to investigate whether *in vitro* exposure to CpG ODNs triggers the proliferation of blood-derived B-CLL (102–104), and whether co-stimuli may make TLR9 signals uniformly stimulatory for B cells. IL-15, an inflammatory cytokine produced by endothelial cells (105, 106), is a plausible candidate for promoting the TLR9-triggered growth of B-CLL. However, this cytokine is best known for its major effects on the growth or survival of NK cells, CD8^+^ T cells, and intra-epithelial γ/σ cells (107, 108). This suggests that the cooperation of CpG ODNs and recombinant human IL-15 may boost the response of B-CLL through TLR9 signaling and the survival of carboxyfluorescein diacetate succinimidyl ester-labeled B-CLL cells with approaches that have yielded important insights concerning clonal growth and the activation-induced death of normal human B cells (109–111).

## Conclusion And Future Perspectives

This review emphasized the immune activity of CpG ODNs with synthetic molecules to produce an innate and adaptive immune system response. Overall, the results show that the incorporation of CpG ODNs and various synthetic molecules improves humoral and cellular immune responses. Thus, the synergistic effects of CpG ODNs enhance both Th1 and Th2 responses and advances maturation of APCs. This review demonstrated the widespread immune response involving CpG and different synthetic molecules such as granulocyte-macrophage colony-stimulating factor (112), IL-15 (113), the STING ligand (114), Gardiquimod (115), IL-10 siRNA (116), poly (I:C) (57, 117, 118), encapsulated poly(γ-PGA-Phe) (119), imiquimod (120, 121), TLR2-neutralizing antibody (95), and iSN34 (47, 48) (Table 1). Furthermore, the application of CpG ODNs and synthetic molecules as adjuvant treatment was demonstrated to improve immunity even in cases with weakened immune systems. Most of the representative studies showed that CpG ODNs and other synthetic molecules induced the immunogenicity of B-cell and effector T-cell responses, macrophage stimulation, and antigen-specific IFN-γ-producing T cells. Therefore, the synergistic activity of CpG DNA and the abovementioned synthetic molecules can induce Th1 cytokine production, thereby stimulating CTLs with increasing Ig production. These activities play a role in the treatment of an extensive spectrum of diseases, comprising cancer, viral and bacterial infections, allergic diseases, and inflammatory disorders (21, 35, 122, 123). Interestingly, these studies also confirmed the clear synergistic activity of TLR9 agonist CpG ODNs and different synthetic molecules (Figure 1B), which may be effective for enhancing immunoregulation in inflammatory disorders, or for heat-killed *Brucella abortus* (HKBa) treatment, antitumor agents, DNA vaccines for B-CLL, measles vaccines, T-cell vaccines, cancer vaccines, and treatment of Lewis lung carcinoma, as well as antiviral action. Patients administered adjuvanted vaccines produced stronger antigen-specific serum antibodies and CD8^+^ and CD4^+^ T-cell responses. Anticancer immunity was produced more quickly in cases immunized with CpG-adjuvanted vaccines (124). However, the immune effects varied, and the effects of CpG ODNs and other immune factors, such as the frequency of NK cells or DCs, were seldom reproducible (100, 125–128). Certainly, demonstrating that the vaccine/adjuvant formulations (synergistic CpG ODNs and synthetic molecules) examined reproducibly change the advancement of tumors is essential. Of greater importance, the immune responses observed barely corresponded to clinical assumptions.

**Table 1:** Synergistic effects of oligodeoxynucleotides combined with synthetic compounds on immunotherapy.

**Synergistic compounds**	**Activated immune response**	**Stimulated cells**	**Regulation of cytokine expression**	**Immunotherapy**	**References**
CpG-ODN +GMCSF	Enhanced Th1 response	CD80 and CD86 stimulation	Induced IL-6 and IL-12	Evaluation in tumor immunization	(112)
CpG-ODN +IL-15	Significant rise in iCa+ followed by rapid apoptosis	CD38^+^ progeny	IL-2, IL-10	Treatment of B-CLL	(113)
CpG-ODN +STING ligand	Th1 response and suppressed Th2	Induced CD8^+^ T cells	IFN-γ, IL-12	Promising antitumor agent	(114)
CpG-ODN +TLR2 neutralizing antibody	Enhanced infiltration of NK cells and CTLs. Reduced type-2 macrophages and Tregs	Production of CD8/CD4	Suppression of TGF-β1, cyclooxygenase-2, and indoleamine 2,3-dioxygenase	Immunotherapeutic against Lewis lung carcinoma	(95)
CpG-ODN (1826) +IL-10 siRNA	Enhanced Th1 response, also induced Th2	CD86, CD80, and CD40 were expressed at a significantly higher percentage on BMDCs	Upregulated IL-12 p35 and IL-12 p40 in BMDCs	DNA vaccine in a mouse model of B-cell lymphoma	(116)
CpG-ODN (2216) +poly I:C (TLR3 ligand)	Enhanced Th1 response	Induced B-cell response	Induced type I-IFN, IL-6, and IL-10	Use as vaccine Potential immunoregulatory function controlling unwanted inflammation	(57, 118)
CpG-ODN +poly I:C (TLR3 ligand) +antigen	Maturation of mAPCs in human immune cells	CD80 and CD86 stimulation	Induced IL-6, type I/II-IFN, IL-12, and TNF-α	Cancer prevention vaccine	(117)
CpG-ODN +γ-PGA-Phe (TLR4 stimulator)	Synergistically activated macrophages and induced Th1 response	Antigen-specific IFN-γ-producing T cells	Induced TNF-α	Vaccine delivery and adjuvant system	(119)
CpG-A +imiquimod (TLR7 ligand)	Promoted memory cell activation linked to DC activation	Induced effector CD8^+^ T-cell responses	Upregulation of IFN-α	T-cell vaccination against infections and malignant diseases	(120, 121)
CpG-ODN (1826) +gardiquimod (TLR7 ligand)	Induced macrophage tolerance	Negative regulation of SOCS1	Reduced TNF-α and IL-6 expression	Impaired response in chronic viral infection	(115)
CpG-ODN (MsST) +iSN34	Enhanced Th1 response	Production of CD19^+^IL-6^+^ cells	Induced IL-6 expression	Prevention or treatment of dysfunction of innate and adaptive immunity	(47)
iSN34 +Pam_3_CSK_4_ (TLR1/2 ligand), LPS (TLR4 ligand), and zymosan (TLR2/6) ligands	Enhanced Th1 response	Production of CD19^+^IL-6^+^ cells	Induced IL-6 expression	Use as agonists and novel therapeutics for inflammatory diseases driven by TLR-mediated immune activation	(48)

*This table lists representative examples of the synergistic activity of ODNs with other molecules and the immunomodulatory activity of the abovementioned immunosynergistic effects*.

*GMCSF, granulocyte macrophage colony-stimulating factor; B-CLL, B-cell chronic lymphocytic leukemia; TGF, transforming growth factor; CTL, cytotoxic T lymphocyte; Treg, regulatory T cell; STING, stimulator of interferon genes; LPS, lipopolysaccharide; poly (I:C), polyinosinic-polycytidylic acid; DC, dendritic cell; IFN, interferon; SOCS1, suppressor of cytokine signaling 1; TNF, tumor necrosis factor; Pam_3_CSK_4_, synthetic triacylated lipopeptide; Th1, T helper type 1*.

Based on the results outlined above, we considered the immunological aspects and ability of vaccine adjuvants to be suitable for use in antitumor immunotherapy, because the mechanisms of activity have been observed *in vitro* and *in vivo*. Additionally, this combination should be evaluated *in vivo* by estimating the initiation of antigen-specific T-cell and B-cell responses after combination immunization in an immunization model. Therefore, our results provide insight into the processes of the associated response of TLR9 and specific synthetic molecules, which potentially encourage the immunotherapeutic and adjuvant equities of their combination.

## Author Contributions

SN and TS conceived, designed, and wrote the manuscript.

### Conflict of Interest Statement

The authors declare that the research was conducted in the absence of any commercial or financial relationships that could be construed as a potential conflict of interest.
